# Educational inequality and COVID-19: Who takes advantage of summer schools and other remedial measures? A latent mediation model analysis based on representative data from Austrian parents of school-aged children

**DOI:** 10.1007/s35834-022-00356-4

**Published:** 2022-09-16

**Authors:** Alexandra Postlbauer, Christoph Helm, Cornelia S. Große

**Affiliations:** 1 Department of Educational Research, Johannes Kepler University Linz, 4040 Linz, Austria; 2 Institute for the Management and Economics of Education, Teacher University Zug, 6300 Zug, Switzerland

**Keywords:** Corona, Social Disparity, Distance Learning, Remedial Interventions, School Closure

## Abstract

In spring 2020, the COVID-19 pandemic triggered a global crisis with far-reaching effects, not least on education. Since the beginning of the pandemic, its impact on learning losses and increasing educational inequality has been widely discussed. While empirical evidence of rising educational inequality and learning loss is steadily growing, at the same time little is known about the families who are interested in remedial measures like summer schools to bridge the negative effects of the pandemic and school closures. The present study addresses this lack of research by providing an initial examination of the empirical evidence of mechanisms underlying parental choice of remedial measures. We take a closer look on which parents are particularly attracted by remedial measures by using cross-sectional data from a parent survey (N = 3590 parents) in Austria. The findings, illustrated via a series of latent mediation models, indicate that parents’ intention to use remedial measures is predicted by parents’ attitudes towards the implementation of remedial measures, parents’ assessment of their child’s learning engagement and of the quality of distance learning during school closures. Moreover, the intention to use remedial measures is significantly influenced by the family’s socioeconomic status.

**Supplementary Information:**

The online version of this article (10.1007/s35834-022-00356-4) contains supplementary material, which is available to authorized users.

## Introduction

School closures and distance learning caused severe negative effects on student achievement (for overviews, see Helm et al. 2021b; König and Frey 2022; Zierer 2021). Learning losses were especially large for children from less-educated or low-income homes, “confirming worries about the uneven toll of the pandemic on children and families” (Engzell et al. 2021, p. 1; see also Helm et al. 2021b). The reasons for both the issues of students’ learning loss and the increase of educational inequality during school closures, are manifold. However, a key driver was the reduction in students’ daily learning time due to the abandonment of regular school structures and the transition to distance education. With respect to learning time, Grätz and Lipps (2021) observed a statistically significant difference of more than four hours per week in the reduction in studying time between secondary students of parents with lower levels of education versus those with higher educational levels during the school closures in the first lockdown. In addition, Grewenig et al. (2020) found a significantly larger reduction in studying time for low-achievers compared to high-achievers. Given the (potentially) negative effects of COVID-19-related school closures, educational policymakers of various countries reacted with remediation strategies to prevent students who were particularly affected by the pandemic from falling (further) behind. Among these strategies, summer schools, holiday care, additional remedial teaching, and emergency support during school closure are discussed (e.g., in Austria: “Corona-Förderpaket für Schülerinnen und Schüler”^1^). While international meta-analyses on effects of summer school programs on various cognitive and non-cognitive outcomes exist^2^, little is known about the effects of remedial strategies implemented in consequence of the COVID-19 pandemic. This is particularly true for mechanisms underlying parents’ intentions to use remedial measures, i.e., sending their children to summer/holiday schools or additional remedial teaching. This is of particular importance, as the socioeconomic dependent usage of remedial measures may, contrary to its initial intention, contribute to inequality (e.g., Schneider 2005). Furthermore, before the COVID-19 pandemic, there were no comparable nationwide remedial programmes in Austria to compensate for learning losses, which were offered throughout the country during non-school hours. Therefore, no findings on the motives and predictors of educational participation in remedial measures are available for Austria and Germany so far (see Klemm and Hollenbach-Biele 2016). In addition, the expansion of the summer school to other subjects as well as the anchoring the summer school in the curriculum of initial teacher education shows that the measure has gained in importance in Austria. Along with this, research into remedial measures is also gaining in importance.

Thus, the purpose of the present study was to identify mechanisms leading to participation or non-participation of children in remedial measures. On the one hand, this gives insights in theoretically expected processes; on the other hand, these insights are of major practical relevance: In order to reduce the gap between children of different socioeconomic status, it is important to know how children in need can be supported, how they can be reached and how they can be motivated to participate in support measures.

## Theoretical framework and empirical findings

### Mechanisms of educational inequality related to educational decision-making

Theoretical explanations for the reproduction of educational inequality are provided in particular by the sociology of education. The concept of primary and secondary effects of origin according to Boudon (1974) is the most popular explanatory approach, on which more recent approaches such as the model of class-specific educational decisions according to Erikson and Jonsson (1996) are based on. The latter model states—in line with the expectancy-value theory by Eccles et al. (1983)—that educational (and achievement-related) decisions are significantly influenced by expectations (of success) and subjective task value.

#### Primary effects of origin

are defined as socialisation processes “that are reflected in class-specific differences in the academic performance and competence of the child” (Becker 2017, p. 115). Socialisation processes refer to a bundle of characteristics of child-rearing that are conducive to learning and that differ significantly between social classes. “Thus, as a result of upbringing, equipment and targeted support in the parental home, children from higher social classes are more likely to acquire skills and motivations that are advantageous in school and education.” (Becker 2017, p. 115).

#### Secondary effects of origin

**Fig. 1:**
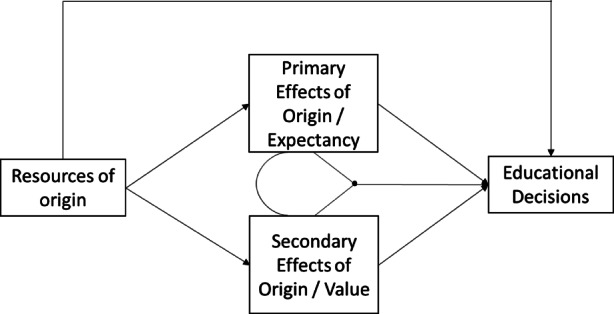
Expectancy-Value Model

(Becker 2017) are social processes that shape the decision-making behaviours of parents. Even if children from different social classes have the same ability (i.e., in the absence of primary effects of origin), parents’ educational decisions differ because the cost-benefit considerations differ according to the social status (e.g., the subjectively expected costs of higher educational qualifications are generally higher for families with few resources of origin). Both, primary and secondary effects of origin directly influence parental educational decision-making (see Fig. 1).

Based on these assumptions, and against the background of the theoretical expectancy-value model of educational decision-making, Esser (1999) argues that class-specific parental educational decisions are based, on the one hand, on differences in the expectation of success at school (e.g., probability of a successful graduation), and, on the other hand, on differences in the value of education (e.g., status maintenance) (see also Eccles et al. 1983; Maaz et al. 2006).

Esser (1999) claims that the e*xpectation of success* at school differs by social class, since the probability of high school performance increases with the membership of an upper social class. The probability of educational progress is higher for upper social classes, as socially privileged students know their way around the system better and are better able to compensate for difficulties. In upper social classes this socialization process, which differs between social classes, is more conducive for the child’s school achievement and motivation. These socialization processes are what Boudon calls the primary effects of origin (Becker 2017).

In addition, Esser (1999) highlights differences in the *value of education* in the form of educational costs and returns depending on the social class. Educational decisions are accompanied by costs, such as a potential loss of status or lower income, and returns, such as the potential access to a certain profession. He assumes that the costs and returns of education are equal for all social classes. However, the loss of status and the associated costs only affect the upper classes. Accordingly, the lower classes need a lesser extent of or no additional education to maintain their status, while this is not the case for upper social classes. Furthermore, lower classes tend to have a lower motivation for education, since for them, the return on education consists only of the return on education itself, while for upper classes, education may also lead to maintaining the social status, since forgoing education would mean a certain loss of status. Moreover, the risk of failure is higher for upper classes (Esser 1999). These class differences in the evaluation of opportunities and risks, or costs and values, of an educational decision are referred to by Boudon as the secondary origin effect (Becker 2017).

Summing up, Esser (1999) explains class-specific educational decisions through the different weighting of educational motivation and cost-return trade-offs per social class, the increased probability of success of upper social classes and their efforts to avert a potential loss of status. Class differences remain even if the costs and returns of education remain constant. The increased probability of success of upper social classes is based on better academic performance as well as on increased financial resources and social relations (*resources of origin*). Grades and teacher recommendations increase or decrease the subjective expectancy of success, but due to class-specific educational motivation they result in different patterns of action.

Educational decision-making can be seen as an interplay of primary and secondary effects of origin. While primary effects of origin predominantly unfold through expectancies of success (due to the performance of the student/child), secondary effects of origin unfold through the value of education in form of class-specific cost-return trade-offs (see Fig. 1).

In the present study, we adopted the expectancy-value model to explain parental decisions for or against the use of remedial interventions (e.g., summer schools). As outlined above, parental decisions can be explained by

- the *expectancies* regarding the remedial measures: For instance, parents with an academic degree are more likely to expect their children to succeed in the remedial measures—due to better academic performance of their children in general.
- the *values* assigned to remedial measures: For instance, parents with an academic degree are more likely to value summer schools higher than parents without an academic degree. Moreover, their efforts to avert a possible loss of status as a result of learning losses are higher. That is, in order to ensure that their own children achieve the same educational status, parents with an academic degree are supposed to make greater efforts—for instance, sending their children to summer school—than parents without an academic degree.

In addition to resources of origin, alternative explanations of parental expectancy and value regarding COVID-19-related remedial measures offered by the educational system may play a significant role. It was assumed that the parental perception of the quality of the distance education and learning and the stress perceived by parents during pandemic had a significant influence on expectancy and value. We assumed that those parents who perceived distance education and learning to be successful were less likely to see a need for remedial measures. Likewise, parents who had hardly perceived any stress during pandemic might have had enough time to support their children and to help them in any school-related respect, thus, they may also not see the need for a remedial measure. Regarding our first assumption, for instance Klemm and Klemm (2010; see also Klemm and Hollenbach-Biele 2016) argue similarly that if parents lose confidence in the individual support of their children by the school, they may rely on compensating for the lack of support in the school learning environment by providing extracurricular learning opportunities and support for their children. Conversely, parents who were satisfied with distance learning, may have had higher confidence in the schooling during the pandemic and therefore rated the remedial measures as less relevant. For working parents, adequate childcare is a central condition for reconciling work and family life. In an Austria-wide parent survey conducted by Lechner et al. (2009), between 8 and 22% of parents stated, depending on the type of childcare, that in addition to the quality and hours of childcare, a lack of alternative childcare (e.g., by grandparents) was a central motive for using school and private childcare services. It is conceivable that the remedial measures in the course of the COVID-19 pandemic were seen by parents as another option for (additional) childcare; especially by parents who experienced the pandemic as particularly stressful, for example due to role and task conflicts (job and childcare).

### Research on remedial measures prior to the COVID-19 pandemic

Remedial measures cover a wide range of support for school children, covering for example private tutoring and summer schools. With respect to private tutoring, in view of the fact that parents pay enormous sums of money to support their children, the question arises how effective these courses are. While a number of studies showed positive effects of tutoring on academic achievement (Berberoğlu and Tansel 2014; Ha and Park 2017), some studies reported mixed or heterogeneous effects (Zhang 2013). In contrast, a number of other studies—in particular from Germany—found no positive effects on academic achievement (Guill and Bos 2014; Hosenfeld 2011; Luplow and Schneider 2014; see Klemm and Hollenbach-Biele 2016 for a discussion). Guill et al. (2020) concluded—based on subsamples of the German National Educational Panel Study (NEPS)—that private tutoring is generally ineffective with regard to enhancing academic achievement; although the authors acknowledged that private tutoring can reduce stress induced by poor performance in school. With respect to the impact of the duration of private tutoring or the qualification of the tutors, in their secondary analysis of two longitudinal studies, Ömeroğulları et al. (2020) found only weak evidence that private tutoring is effective; specifically, they found neither positive effects of a longer duration of tutoring nor of a higher qualification of the tutors. However, they concluded that for specific combinations of prior knowledge, tutor qualifications, and school subjects, private tutoring may have positive effects. Just recently, Guill et al. (2021) were also not able to identify positive effects of private tutoring on school achievement.

While private tutoring is—as the name already suggests—organized privately, in some cases remedial measures are also organized by schools or educational authorities, for example in the form of summer schools. In their meta-analysis, Kidron and Lindsay (2014) arrived at the conclusion that increased learning time enhanced achievement when the courses are conducted by certified teachers; however, the effects were small. Patall et al. (2010) analysed the effects of extending school days or school years, and they concluded that extending school time can foster learning outcomes, especially for students most at risk of school failure. Furthermore, Dietrichson et al. (2017) found a small but positive average effect size of 0.03 (not statistically significant) for summer programs (referring to eight unique study samples). Ritter et al. (2009) reported positive effects for students working with volunteer tutors on various achievement measures.

Based on these considerations the question arises who actually participates in tutoring. Does taking part in private tutoring or summer schools depend on socioeconomic factors or the educational level of the parents? And do the effects of private tutoring or summer schools depend on these aspects as well?

Based on a sample of students in India, Dongre and Tewary (2015) reported a positive effect of private tutoring on achievement, and this effect was more pronounced for disadvantaged students; thus, less wealthy students with less educated parents benefitted even more from private tutoring. However, in this study, the students spent on average nine hours per week in tuitions, raising study time substantially (and probably to a considerably higher extent compared to the average duration of private tutoring in Austria or Germany). Based on questionnaire data from parents in South Korea, Kim and Park (2010) found that household income and parents’ educational level were positively associated with a higher amount of private tutoring. Similarly, and also with respect to South Korea, Jung and Lee (2010) found positive correlations between private tutoring (both with respect to participation and expenditures) and the mother’s educational attainment and the household income, while the mother’s employment status correlated negatively with private tutoring. This latter effect was more salient for non-professional working women (compared to professional working women), and it was explained with time constraints of working mothers (and, in case of non-professional working mothers, with a lower priority they place on private tutoring).

With respect to elementary schools in Germany, Luplow and Schneider (2014) concluded that private tutoring does not increase social disparities, as it is neither used more often by privileged groups, nor does it foster the development of competencies. In another German study, Abele and Liebau (1998) also found no income effect on private tutoring consumption. In contrast, Schneider (2005) found that tutoring is increasingly used as household income rises. In a Swiss study, Hof and Wolter (2014) showed that approximately one third of all 8th and 9th grade students took private tutoring. Two thirds of them participated on a regular basis, and they came particularly often from socially privileged parental homes. However, the effects of private tutoring were small, especially in case of regular private tutoring. Even a negative impact is possible, if students’ self-regulated study time is replaced by (less effective) private tutoring. In their study, Hof and Wolter (2014) found that, independently of gender, age, migration background or competence level of the students, the educational level of the parents significantly influenced whether private tutoring was attended. Thus, given equal competencies and otherwise comparable conditions, the participation in tutoring depends on the parents’ educational levels. In addition, the socioeconomic status is of importance: the lower the occupational status of the parents, the less often tutoring is attended. Consequently, Hof and Wolter (2014) concluded that this suggests that equity of opportunity may be violated.

### Research on COVID-19-related remedial measures

COVID-19-related school closures and their effects on students’ learning have been researched intensively (see for example Helm et al. 2021a, for an overview on findings in Germany, Austria, and Switzerland, and Helm et al. 2021b, for an overview on learning losses and educational inequalities). In addition, several studies focused on students’ learning time during lockdowns (Dietrich et al. 2020; Grätz and Lipps 2021; Grewenig et al. 2020; Huber and Helm 2020; Züchner and Jäkel 2021). At student level, the findings indicated that learning time was positively predicted by age (with older students spending more time on learning), performance, diligence, and positive emotions during school closure. At the contextual level, school type (with academic track students spending more time for learning), instructional quality, social support, and home/family resources positively predicted students’ learning effort. In addition, several studies (Huber et al. 2020; Porsch and Porsch 2020; Tengler et al. 2020; Wacker et al. 2020; Wößmann et al. 2020) reported ranges of up to 60% of students spending a maximum of two hours a day on school-related activities during school closure.

To compensate for learning time losses, Austrian education policy (like many other countries) relies on remedial offers (e.g., additional teaching and summer school). In the context of COVID-19, potential remediation strategies were first discussed and compared by Pan and Sass (2020). They concluded that lengthening the school year by two weeks produced only moderate improvements, while lengthening school days and summer school programs could reduce learning losses substantially. In Germany and Austria, summer school programs are offered in order to make up for missed learning. Wößmann et al. (2021) reported that 21% of the students in Germany participated in tutoring to make up for missed school lessons after the first school closures. Specifically, however, low-achieving children did not attend summer schools or remedial classes more often than high-achieving children, although these offers were intended primarily for them. In addition, children from educationally disadvantaged families received particularly little support in terms of remedial measures such as tutoring. This is a highly interesting finding since it points to potential additional increases in educational inequality. Unfortunately, Wößmann et al. (2021) did not provide an explanation for this unexpected finding. We think that a possible explanation could be that intended and non-intended effects balance each other out here. That is, under “intended effects” we would expect a higher participation among lower-performing students. Under “non-intended effects”, we would expect—due to the secondary origin effect (see above)—higher participation among high-achieving pupils, who mostly come from socially better-off and educationally closer families. Furthermore, it is also conceivable that for parents of all children (regardless of whether they are high achievers or not), the primary motive for using remedial measures may lie in additional childcare and less in compensating for learning losses.

In this paper we provide an initial examination of the empirical evidence of mechanisms underlying parental choice of remedial measures. We take a closer look at which parents are particularly attracted by remedial measures.

## Research questions

Against the background of existing research findings indicating that socially disadvantaged families are less likely to use remedial measures (Hof and Wolter 2014; Jung and Lee 2010; Kim and Park 2010; Wößmann et al. 2021) or at least not to a higher extent (Luplow and Schneider 2014), we assumed a positive relationship between indicators of social and cultural origin on the one hand, and parental intention to use remedial measures on the other hand. Furthermore, we assumed (in alignment with the expectancy-value theory; Eccles et al. 1983; Esser 1999; Maaz et al. 2006) that social and cultural origin are indirectly related to parents’ intention via parents’ expectancy of success and value.

**Fig. 2:**
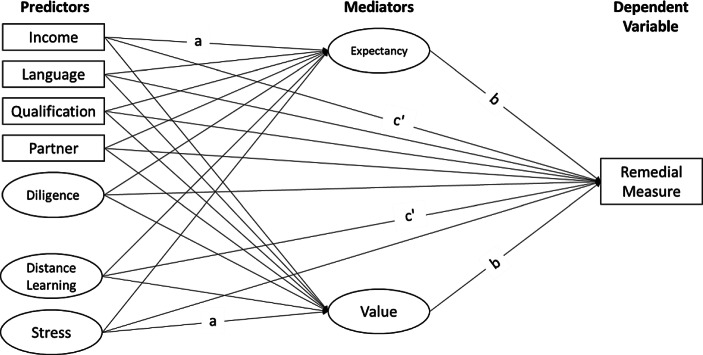
Latent mediation model*. *(*Income* Monthly net household income, *Language* Language spoken at home, *Qualification* Parents’ highest educational level, *Partner* Single-parent household status, *Diligence* Scholastic diligence, *Distance Learning* Quality of distance learning, *Stress* Parental stress. *a* refers to all paths between predictors and mediators, *b* refers to all paths between mediators and dependent variable, and *c’* refers to all paths between predictors and dependent variable with mediators. Interaction effects are not shown)

More concretely, we tested a *mediation model* that illuminates underlying mechanisms of parental intention to use remedial measures (see Fig. 2). The model was specified as follows: Parents’ intention to use remedial measures (i.e., summer school, additional tutoring, tutoring during the semester break, tutoring during the easter break) represented the dependent variable. Indicators of social origin (monthly net household income, parents’ highest educational level, single-parent household status), cultural origin (language spoken at home), and scholastic diligence were predictors. Additionally, we included quality of distance learning and parental stress as further predictors to control for central aspects of distance learning. We did so, as quality of distance learning and perceived stress are supposed to significantly influence students learning outcomes during COVID-19 (e.g., Helm and Huber 2022). Those parents who considered distance learning to be successful or who had hardly perceived any stress are most likely to see no need for remedial measures. Finally, parents’ expectancy of success and parents’ value regarding remedial measures were specified as mediators.

In particular, we assumed that parents who score high on the expectancy and value component should be more likely to use the remedial measures than parents who score high on only one of the two components. That is, parents who thought that the remedial measures would be successful and who had positive attitudes towards the measures were more likely to also respond affirming to the question on whether they will use the remedial measures.

## Research design

### Sample and data collection process

To test our hypotheses, we used data from a representative parent survey (*N* = 3590 parents, 70.6% female, 37.7% with children from primary school, 37.9% from lower secondary school, 24.3% from upper secondary school) that was carried out after the third lockdown in Austria at the beginning of 2021. Austrian parents of children attending compulsory school represented the target group of the online questionnaire. To obtain a representative sample, the parent survey was carried out by a market research institute and, additionally, by a parent association. Furthermore, we used Iterative Proportional Fitting (also called raking) as a post-stratification procedure to ascribe weights to the data against the background of official statistics (Eurostat 2020; Statistics Austria 2020) regarding the following variables: child’s sex, school type, parents’ highest educational level, monthly net household income, employment status before COVID-19, language spoken at home, number of children in the household, single-parent household status, community size, and federal state.

The median monthly net household income was 3500 euros. 127 of the families spoke a non-German language at home. The following languages were reported more than three times: English (30), Turkish (15), Bosnian (9), Hungarian (8), Spanish (7), Serbian (5), Romanian (5), Italian (5), French (5), Albanian (4), Russian (4), Polish (4), Slovak (4). 33.9% of the parents interviewed had an academic degree, 29.4% completed secondary school education, 20.4% made an apprenticeship and 1.3% had only a compulsory school leaving certificate. The remaining 15% had other school-leaving qualifications. 11.6% lived in a single-parent household.

### Measures

#### Outcome

To capture the parental intention to use remedial measures (outcome variable), parents were asked to indicate whether they intend to use a summer school, additional tutoring, tutoring during the semester break, or tutoring during the easter break. Parents’ intention to use the respective remedial measure was assessed by no = 0, not sure = 1, and yes = 2.

#### Predictors

To assess resources of origin (i.e., students’ socioeconomic and cultural background), parents were asked to provide information on their highest education, monthly net household income, language spoken at home, and whether they were a single parent. These indicators were used separately, because we assumed that they exert differential effects on the mediating and the dependent variables of our study. In addition, based on research about COVID-19-related school closures (e.g., Huber and Helm 2020; Wößmann et al. 2020), we selected items referring to scholastic diligence (e.g., ‘My child is hardworking.’), quality of distance learning (e.g., ‘How high do you rate the quality of your child’s distance education during the COVID-19-related school closures?’) and parental stress (e.g., ‘I hardly have time for myself.’).

#### Mediators

Finally, we asked for parents’ expectation of success (e.g., ‘It is planned that the additional learning support during the vacations will be mainly provided by student teachers. Do you think that they will be able to give sufficient consideration to the needs of your child?’), and value of the measures (e.g., ‘Are you in favour of or against the implementation of additional remedial teaching for weaker pupils?’).

Parents rated all items on a five-point Likert scale, with response categories ranging from (1) “strongly disagree”/“low” to (5) “strongly agree”/“high”. See Table 1 for the psychometric properties of the scales, descriptive statistics and reliability indices, and Table 5 in the Appendix for a full correlation matrix of the study variables.

**Table 1:** Descriptive Statistics

	*#*	$\mathrm{M}$	*SD*	Min	Max	α
Summer School	1	0.806	0.883	0	2	–
Additional Tutoring	1	1.149	0.877	0	2	–
Tutoring during the Semester Break	1	0.645	0.645	0	2	–
Tutoring during the Easter Break	1	0.655	0.864	0	2	–
Income	1	3,746.994	1,686.165	350	15,000	–
Language	1	1.036	0.186	1	2	–
Qualification	1	2.602	1.638	1	8	–
Partner	1	1.116	0.320	1	2	–
Diligence	2	2.241	1.060	–	–	0.875
Distance Learning	2	2.947	1.011	–	–	0.725
Stress	2	3.311	1.304	–	–	0.860
Expectancy 1^a^	1	2.861	0.955	–	–	–
Expectancy 2^a^	1	3.367	1.358	–	–	–
Value	3	4.256	0.095	–	–	0.890

^a^The items on expectancy are reported individually due to different response formats

*#* number of items, *M* mean, *SD* standard deviation, *α* Cronbach’s alpha, *Income* Monthly net household income, *Language* Language spoken at home, *Qualification* Parents’ highest educational level, *Partner* Single-parent household status, *Diligence* Scholastic diligence, *Distance Learning* Quality of distance learning, *Stress* Parental stress

### Analytical procedure

#### Model specification

To investigate direct and indirect effects of students’ socioeconomic and cultural status, scholastic diligence, and central aspects of distance learning (quality of the learning environment, parental stress) on parents’ intention to use remedial measures, we performed structural equation models. More concretely, we performed a separate latent mediation analysis (as displayed in Fig. 2) for four remedial measures.

#### Model estimation

For predicting categorical dependent variables we used the WLSMV estimator. To obtain unbiased *p*-values for the indirect effects, we used bias-corrected bootstrapping with 1000 iterations. Bias-corrected bootstrapping represents a non-parametric resampling method (instead of an asymptotic strategy like the Sobel test (Sobel 1982), also called the product-of coefficients approach, which are based on the standard normal distribution)—see Preacher and Hayes (2008) for further details. More concretely, the bias-corrected bootstrapping method “corrects for bias in the central tendency of the estimate.” (MacKinnon et al. 2004, p. 115). The bias is expressed by “z score of the percentile of the observed sample indirect effect.” (MacKinnon et al. 2004, p. 115). The formula for calculating the lower and upper confidence limits is given in MacKinnon et al. (2004, p. 115). Bootstrap confidence intervals are based on an empirical estimation of the sampling distribution of the indirect effect; they can be asymmetrical (Preacher and Hayes 2008).

#### Model evaluation

To determine the model fit, we used common cut-off criteria (Hu and Bentler 1999; Little 2013)—Bentler’s comparative fit index (CFI ≥ 0.90), the Tucker-Lewis index (TLI ≥ 0.90), the root mean square error of approximation (RMSEA ≤ 0.08), and the weighted root mean square residual (WRMR ≤ 1.00).

#### Missing data

All the variables used have missing values. Few items have more than 6% missing values (14% household income, 20% attitude towards special support classes in Math, 29% attitude towards special support classes in German). Subsequent analyses applied the Full Information Maximum Likelihood (FIML) method. ‘In this method, all students are included for an analysis and the missing data is “integrated out”’ (Robitzsch et al. 2016, pp. 290–291). A central assumption of the FIML procedure is that, after controlling for all variables in the analysis, missing values are randomly distributed (i.e., independent of the levels of other variables in the analysis). Since our analyses include parents’ socioeconomic background and their children’s engagement, we considered variables that are supposed to be predictive for missing student data.

#### Software used

All analyses were conducted using the R package ‘MplusAutomation’ (Hallquist and Wiley 2016) in combination with Mplus 8 (Muthén & Muthén 1998–2017).

## Results

### Descriptive, bivariate statistics

showed that single parents, low-income earners (exception: summer school), families who do not speak German at home and non-academics tended to express interest in the four remedial offers more often than other parents (see Figs. 7, 8, 9 and 10 in the Appendix).

### Inferential and multivariate statistics,

**Fig. 3:**
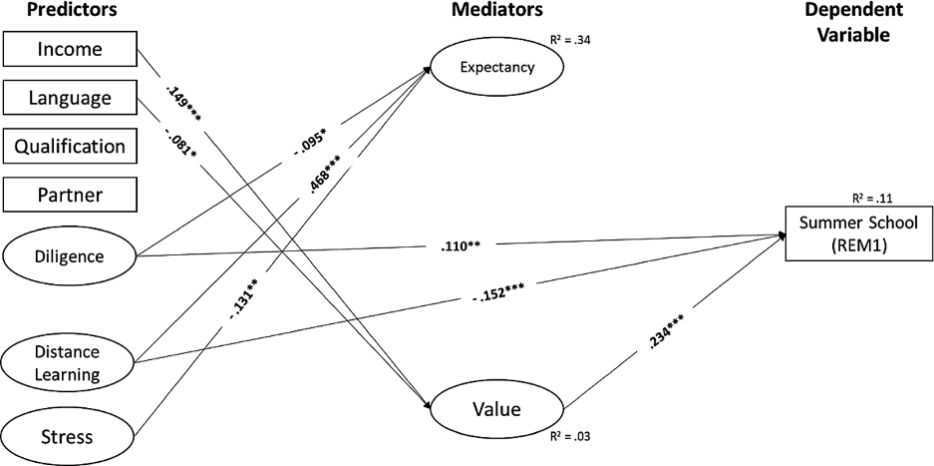
Summer School (*REM1*). (Only statistically significant effects are reported. *Income* Monthly net household income, *Language* Language spoken at home, *Qualification* Parents’ highest educational level, *Partner* Single-parent household status, *Diligence* Scholastic diligence, *Distance Learning* Quality of distance learning, *Stress* Parental stress)

**Fig. 4:**
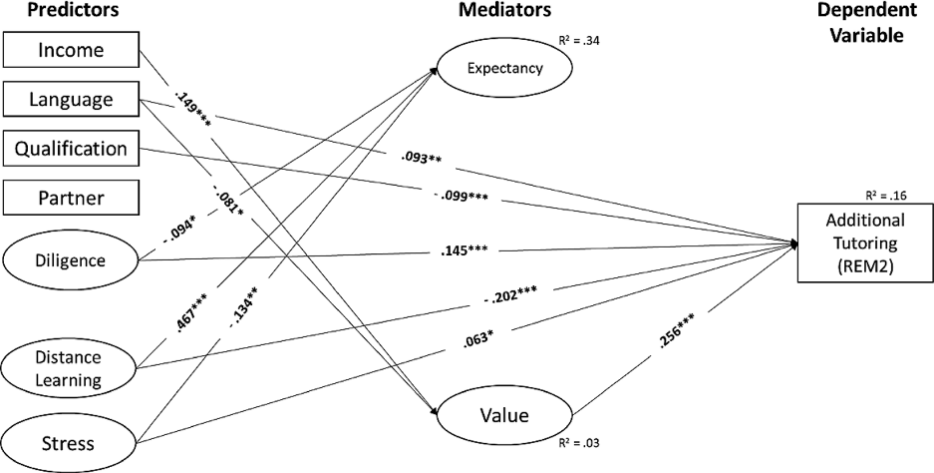
Additional Tutoring (*REM2*). (Only statistically significant effects are reported. *Income* Monthly net household income, *Language* Language spoken at home, *Qualification* Parents’ highest educational level, *Partner* Single-parent household status, *Diligence* Scholastic diligence, *Distance Learning* Quality of distance learning, *Stress* Parental stress)

**Fig. 5:**
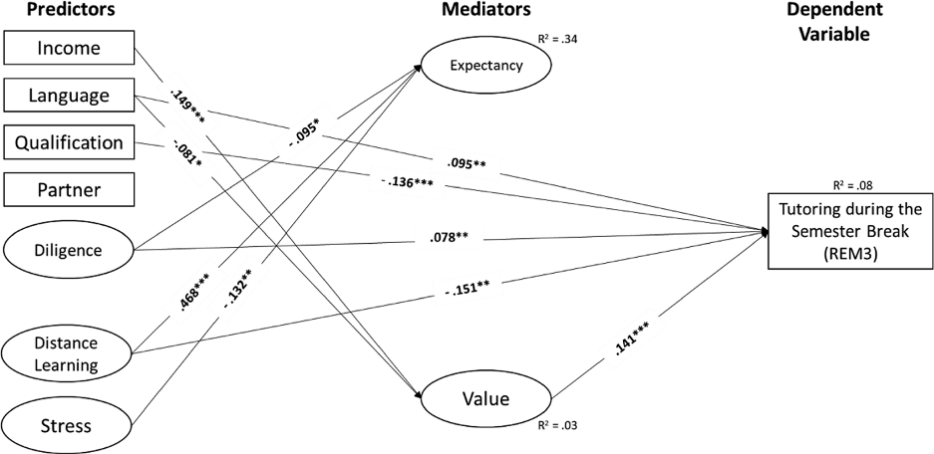
Tutoring during the Semester Break (*REM3*). (Only statistically significant effects are reported. *Income* Monthly net household income, *Language* Language spoken at home, *Qualification* Parents’ highest educational level, *Partner* Single-parent household status, *Diligence* Scholastic diligence, *Distance Learning* Quality of distance learning, *Stress* Parental stress)

**Fig. 6:**
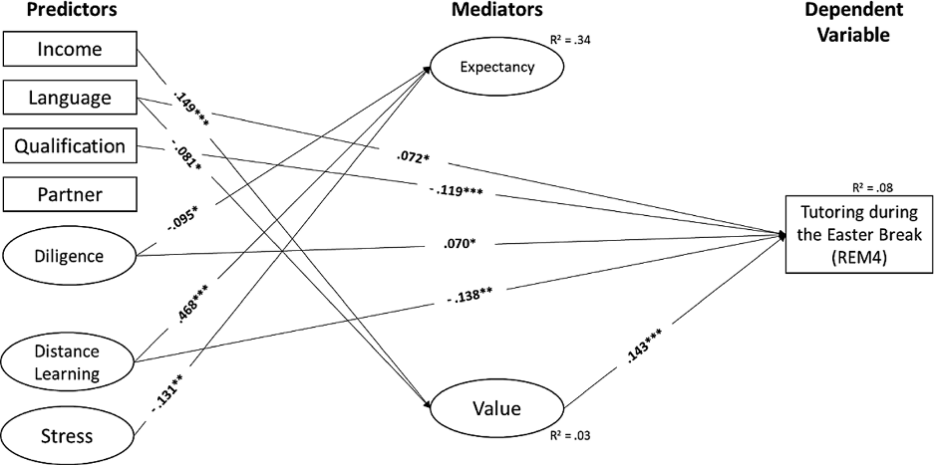
Tutoring during the Easter Break (*REM4*). (Only statistically significant effects are reported. *Income* Monthly net household income, *Language* Language spoken at home, *Qualification* Parents’ highest educational level, *Partner* Single-parent household status, *Diligence* Scholastic diligence, *Distance Learning* Quality of distance learning, *Stress* Parental stress)

**Fig. 7:**
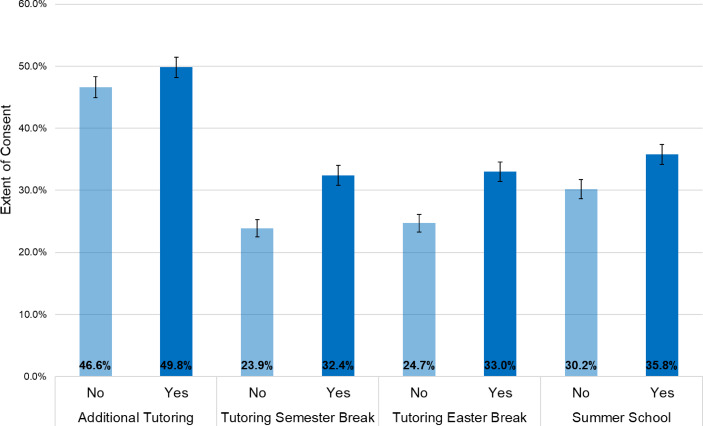
Extent of consent to different remedial offers—Single Parent Household. (Error bars represent the 95% confidence interval. An overlap of the intervals of two bars indicates a non-significant difference)

**Fig. 8:**
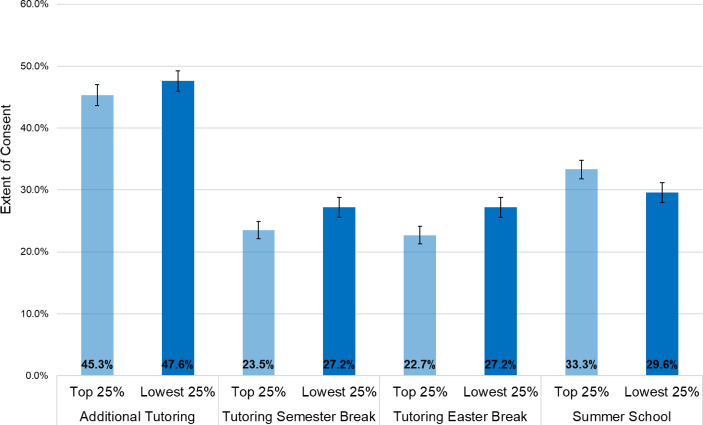
Extent of consent to different remedial offers—Household Income. (Error bars represent the 95% confidence interval. An overlap of the intervals of two bars indicates a non-significant difference)

**Fig. 9:**
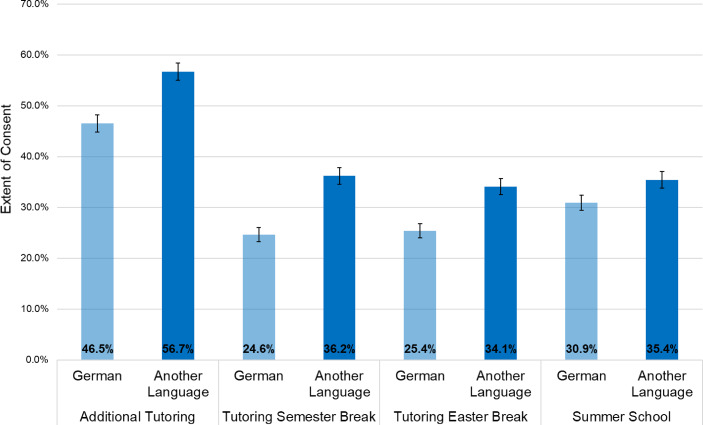
Extent of consent to different remedial offers—Language spoken at home. (Error bars represent the 95% confidence interval. An overlap of the intervals of two bars indicates a non-significant difference)

**Fig. 10:**
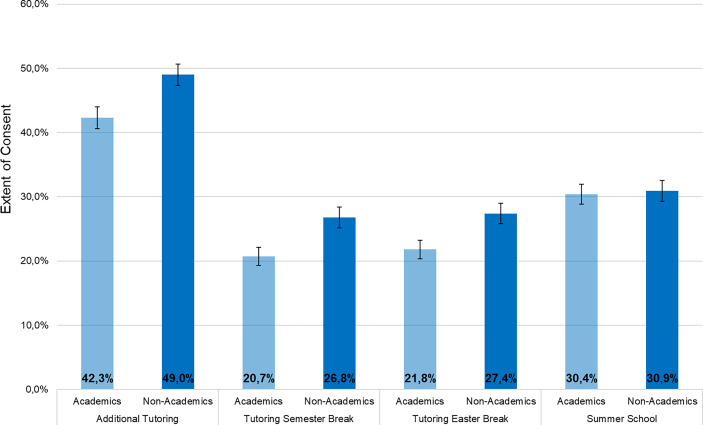
Extent of consent to different remedial offers—Parental Education. (Error bars represent the 95% confidence interval. An overlap of the intervals of two bars indicates a non-significant difference)

i.e., structural equation modelling, showed that the overall fits of our hypothesized models were good (see Table 2), indicating that our proposed mediation models reasonably accounted for the observed data. Moreover, across all latent mediation models, we found the following empirical support for the hypothesized direct (a, b, c’) and indirect (a * b) paths (see Fig. 2 and more specifically Figs. 3, 4, 5 and 6, as well as Table 4 in the Appendix).

**Table 2:** Model fit indices

Model	N	Par	χ^2^	*df*	*p*	χ^2^/*df*	CFI	TLI	RMSEA	WRMR	R^2^
REM1	3582	93	236.100	59	< 0.001	4.00	0.955	0.909	0.029 [0.025, 0.033]	0.834	0.11
REM2	3582	93	175.002	59	< 0.001	2.97	0.971	0.942	0.023 [0.019, 0.027]	0.710	0.16
REM3	3582	93	235.894	59	< 0.001	4.00	0.955	0.908	0.029 [0.025, 0.033]	0.834	0.08
REM4	3582	93	229.576	59	< 0.001	3.89	0.956	0.911	0.028 [0.025, 0.032]	0.822	0.08

*REM* remedial measure, *N* sample size, *Par* Number of parameters estimated, *χ*^*2*^ Chi square value, *df* degrees of freedom, *p* p value, *CFI* Bentler’s comparative fit index, *TLI* Tucker-Lewis index, *RMSEA* root mean square error of approximation, *WRMR* weighted root mean square residual, *R*^*2*^ r square

### Predictors of parents’ intention to use remedial measures (direct effects)

Regarding the predictive power of expectancy and values (i.e., the *b‑paths*), we only found a significant positive association between parents’ attitude towards implementing remedial measures (value) and their intention to use them (β_REM1_ = 0.234, *p* < 0.001; β_REM2_ = 0.256, *p* < 0.001; β_REM3_ = 0.141, *p* < 0.001; β_REM4_ = 0.143, *p* < 0.001). In contrast, we found that parents’ expectation of success with regard to remedial measures did not significantly influence their planned use of the measures (β_REM1_ = −0.018, *p* = 0.763; β_REM2_ = 0.055, *p* = 0.368; β_REM3_ = −0.001, *p* = 0.988; β_REM4_ = −0.035, *p* = 0.584).

Regarding the predictive power of resources of origin and perceived quality of distance learning and parental stress (i.e., the *c’-paths*), our results showed that the higher parents rated their children’s learning engagement in general (β_REM1_ = 0.110, *p* < 0.001; β_REM2_ = 0.145, *p* < 0.001; β_REM3_ = 0.078, *p* = 0.006; β_REM4_ = 0.070, *p* = 0.016), and the lower parents rated the quality of distance learning during COVID-19-related school closures (β_REM1_ = −0.152, *p* < 0.001; β_REM2_ = −0.202, *p* < 0.001; β_REM3_ = −0.151, *p* = 0.001; β_REM4_ = −0.138, *p* = 0.002), the more likely were parents’ intentions to use remedial measures.

In addition, for all remedial measures except “summer school”, parents were more likely to indicate their intention to use tutoring during the semester break, the lower their level of qualification (β_REM2_ = −0.099, *p* < 0.001; β_REM3_ = −0.136, *p* < 0.001; β_REM4_ = −0.119, *p* < 0.001) and if they speak German at home (β_REM2_ = 0.093, *p* = 0.002; β_REM3_ = 0.095, *p* = 0.001; β_REM4_ = 0.072, *p* = 0.016).

### Predictors of parents’ expectancy and value regarding remedial measures (direct effects)

Regarding the factors affecting parents’ expectations and values regarding remedial measures (i.e., the *a‑paths*), we found that parents with a higher income were more likely to be in favour of remedial measures (β_REM1_ = 0.149, *p* < 0.001; β_REM2_ = 0.149, *p* < 0.001; β_REM3_ = 0.149, *p* < 0.001; β_REM4_ = 0.149, *p* < 0.001). In contrast, parents who speak a language other than German at home were more likely to be against remedial measures (β_REM1_ = −0.081, *p* = 0.019; β_REM2_ = −0.081, *p* = 0.019; β_REM3_ = −0.081, *p* = 0.019; β_REM4_ = −0.081, *p* = 0.019). Furthermore, parents who rated the quality of distance learning during COVID-19-related school closures higher had higher expectations about the success of remedial interventions (β_REM1_ = 0.468, *p* < 0.001; β_REM2_ = 0.467, *p* < 0.001; β_REM3_ = 0.468, *p* < 0.001; β_REM4_ = 0.468, *p* < 0.001). The lower parents rated their children’s learning engagement in general (β_REM1_ = −0.095, *p* = 0.012; β_REM2_ = −0.094, *p* = 0.014; β_REM3_ = −0.095, *p* = 0.013; β_REM4_ = −0.095, *p* = 0.012) and the less they were stressed during COVID-19-related school closures (β_REM1_ = −0.131, *p* = 0.002; β_REM2_ = −0.134, *p* = 0.001; β_REM3_ = −0.132, *p* = 0.002; β_REM4_ = −0.131, *p* = 0.002), the higher their expectations about the success of remedial measures.

### Indirect effects of resources of origin

Regarding the indirect standardized effects (a * b‑paths) (see Table 3), we found a significant impact of monthly net household income (indirect std. effect_REM1_ = 0.035, 95%-CI [0.023, 0.054]; indirect std. effect_REM2_ = 0.038, 95%-CI [0.024, 0.055]; indirect std. effect_REM3_ = 0.021, 95%-CI [0.012, 0.035]; indirect std. effect_REM4_ = 0.021, 95%-CI [0.011, 0.034]) and German language spoken at home (indirect std. effect_REM1_ = −0.019, 95%-CI [−0.038, −0.004]; indirect std. effect_REM2_ = −0.021, 95%-CI [−0.041, −0.004]; indirect std. effect_REM3_ = −0.011, 95%-CI [−0.025, −0.003]; indirect std. effect_REM4_ = −0.012, 95%-CI [−0.025, −0.003]) on parents’ intentions to use remedial measures via parents’ valuation of the respective remedial measure (value component). These indirect effects were true across all four remedial measures.

**Table 3:** Indirect effects

**Summer School (REM1)**						
*–*	*Predictor*	*Intervening*	*Outcome*	*Std. estimate*	*Low 2.5*	*Up 2.5*
1	Diligence	Expectancy	REM1	0.002	−0.009	0.018
2	Diligence	Value	REM1	−0.006	−0.018	0.006
3	Distance learning	Expectancy	REM1	−0.009	−0.062	0.051
4	Distance learning	Value	REM1	0.007	−0.008	0.023
5	Stress	Expectancy	REM1	0.002	−0.014	0.019
6	Stress	Value	REM1	0.007	−0.006	0.019
7	Language	Expectancy	REM1	0.001	−0.003	0.014
8	Language	Value	REM1	−0.019	−0.038	−0.004
9	Income	Expectancy	REM1	−0.001	−0.011	0.005
10	Income	Value	REM1	0.035	0.023	0.054
11	Qualification	Expectancy	REM1	−0.001	−0.012	0.007
12	Qualification	Value	REM1	−0.005	−0.016	0.006
13	Partner	Expectancy	REM1	0.001	−0.003	0.009
14	Partner	Value	REM1	0.004	−0.007	0.014
**Additional Tutoring (REM2)**						
*–*	*Predictor*	*Intervening*	*Outcome*	*Std. estimate*	*Low 2.5*	*Up 2.5*
1	Diligence	Expectancy	REM2	−0.005	−0.026	0.004
2	Diligence	Value	REM2	−0.007	−0.020	0.006
3	Distance learning	Expectancy	REM2	0.025	−0.026	0.090
4	Distance learning	Value	REM2	0.008	−0.009	0.024
5	Stress	Expectancy	REM2	−0.007	−0.031	0.006
6	Stress	Value	REM2	0.008	−0.006	0.021
7	Language	Expectancy	REM2	−0.002	−0.016	0.002
8	Language	Value	REM2	−0.021	−0.041	−0.004
9	Income	Expectancy	REM2	0.003	−0.002	0.016
10	Income	Value	REM2	0.038	0.024	0.055
11	Qualification	Expectancy	REM2	0.004	−0.003	0.019
12	Qualification	Value	REM2	−0.005	−0.018	0.008
13	Partner	Expectancy	REM2	−0.002	−0.012	0.001
14	Partner	Value	REM2	0.004	−0.008	0.015
**Tutoring during the Semester Break (REM3)**						
*–*	*Predictor*	*Intervening*	*Outcome*	*Std. estimate*	*Low 2.5*	*Up 2.5*
1	Diligence	Expectancy	REM3	0.000	−0.013	0.015
2	Diligence	Value	REM3	−0.004	−0.012	0.003
3	Distance learning	Expectancy	REM3	0.000	−0.057	0.061
4	Distance learning	Value	REM3	0.004	−0.005	0.014
5	Stress	Expectancy	REM3	0.000	−0.020	0.016
6	Stress	Value	REM3	0.004	−0.003	0.012
7	Language	Expectancy	REM3	0.000	−0.008	0.008
8	Language	Value	REM3	−0.011	−0.025	−0.003
9	Income	Expectancy	REM3	0.000	−0.008	0.007
10	Income	Value	REM3	0.021	0.012	0.035
11	Qualification	Expectancy	REM3	0.000	−0.010	0.011
12	Qualification	Value	REM3	−0.003	−0.010	0.004
13	Partner	Expectancy	REM3	0.000	−0.005	0.006
14	Partner	Value	REM3	0.002	−0.004	0.009
**Tutoring during the Easter Break (REM4)**						
*–*	*Predictor*	*Intervening*	*Outcome*	*Std. estimate*	*Low 2.5*	*Up 2.5*
1	Diligence	Expectancy	REM4	0.003	−0.008	0.020
2	Diligence	Value	REM4	−0.004	−0.013	0.003
3	Distance learning	Expectancy	REM4	−0.016	−0.072	0.047
4	Distance learning	Value	REM4	0.004	−0.004	0.015
5	Stress	Expectancy	REM4	0.005	−0.013	0.023
6	Stress	Value	REM4	0.004	−0.003	0.012
7	Language	Expectancy	REM4	0.001	−0.002	0.015
8	Language	Value	REM4	−0.012	−0.025	−0.003
9	Income	Expectancy	REM4	−0.002	−0.014	0.004
10	Income	Value	REM4	0.021	0.011	0.034
11	Qualification	Expectancy	REM4	−0.002	−0.015	0.006
12	Qualification	Value	REM4	−0.003	−0.011	0.004
13	Partner	Expectancy	REM4	0.001	−0.002	0.011
14	Partner	Value	REM4	0.002	−0.005	0.009

*REM* remedial measure, *Diligence* Scholastic diligence, *Distance Learning* Quality of distance learning, *Stress* Parental stress, *Qualification* Parents’ highest educational level, *Income* Monthly net household income, *Language* Language spoken at home, *Partner* Single-parent household status

### Latent interaction effects of the expectancy and value components

To test for the *interaction effect,* we modelled a latent interaction between the *expectancy and value *components using the XWITH command in Mplus. However, for three of four models the results indicated non-significant interaction coefficients. Thus, our hypotheses were rejected (REM1: std. beta = −0.042, *p* = 0.155; REM3: −0.023, *p* = 0.448; REM4: −0.056, *p* = 0.067). Only model 2 was statistically significant (REM2: −0.075, *p* = 0.007).

## Discussion

While empirical evidence of rising educational inequality and learning loss is steadily growing (Helm et al. 2021b), at the same time little is known about which families are interested in remedial measures like summer schools to bridge the negative effects of the pandemic and the accompanying school closures. In other words, the question of whether and to what extent socioeconomic origin influences participation in such remedial measures—and thus may reflect inequality—has remained largely unresolved. The present study addressed this lack of research by providing an initial examination of the empirical evidence of mechanisms underlying parental choice of remedial measures. We took a closer look at which groups of parents were particularly attracted by remedial measures by using cross-sectional data from a parent survey in Austria. Descriptively, the findings showed that single parents, families who do not speak the national language at home, and non-academics tended to express interest in remedial offers more often than other parents. For low-income earners, the results showed the same trend except for summer schools, which are appreciated less compared to higher-income families. Thus, it can be surmised that these remedial offers largely reached the target group.

In contrast, however, according to the expectancy-value model of educational decision-making by Esser (1999), the mediation analyses showed that parents with higher income tended to use these measures more, while non-German speaking parents tended to oppose these measures. These associations were mediated by parents’ attitudes towards the remedial measures (i.e., value component), but, contrary to expectations, not by parents’ expectations (i.e., expectancy component). These findings can also be interpreted within the framework of rational choice theory, to which the model can be attributed. Accordingly, parents with a higher level of education and a higher social status were more concerned that their children maintain the social status. Therefore, they had a greater interest than less educated parents in avoiding or compensating for any learning losses of their children, hence they placed greater value on remedial measures. Another explanation could be that less educated parents knew less about learning losses and their potential long-term negative consequences. Mechanisms underlying these contradictory effects, like status maintenance and the importance of knowledge about the long-term consequences of educational decisions, should be further investigated in future studies.

In addition, the results showed a significant positive impact of household income and German language spoken at home on parents’ intention to use remedial measures via parents’ valuation of these measures. This result indicates a social inequality that is already manifested in the perceived value of such measures. These findings are also in line with expectancy-value theoretical model of educational decision-making by Esser (1999).

Against the background of expectancy-value theory (Eccles et al. 1983; Esser 1999; Maaz et al. 2006) we found that parents with a positive attitude towards remedial measures intended to use them more often; in contrast, parents’ expectations of these measures’ success did not significantly influence their planned use of the measures. At least in part, this effect can be explained by considering parents who may agree on benefits of remedial measures in general, but who do not recognize the benefit or need for their own children. However, if one also considers the contention that actually parents with no “need” for remedial measures for their children (i.e., children with high levels of scholastic diligence) intended to use remedial measures, the impression arises that the actual target group of remedial measures cannot be reached in a comprehensive way. However, it should be noted that at the time of the survey, the parents were not yet informed by the schools whether it would be particularly advisable for their children to actually participate in summer schools. Thus, surveys conducted later in the school year might have provided more differentiated results.

The quality of distance learning during school closure was also relevant for parents’ intentions to use remedial measures. As expected, the lower the perceived quality of distance learning, the higher the intention to take advantage of remedial offers. In this case, one can indeed speak of “compensatory” measures if the actual aim is to compensate for a lack of learning opportunities.

Contrary to expectations, the interaction effect between the expectancy- and the value-components was negative. That is, parents who thought that the remedial measures would be successful and who had positive attitudes towards the measures were less likely to use additional tutoring. One explanation for the lack of effects is the missing association between the two components. Table 5 shows that the items of the expectancy and value components were non-significantly and only very weakly correlated. That is, higher values in one component did not go hand in hand with higher values in the other component. Thus, an interaction effect was unlikely. From a content-specific point of view, one could explain missing interaction effects as follows. When looking at the item text: (1) Items of the value component asked whether parents were in favor or against the implementation of remedial measures; (2) the items of the expectancy component asked whether parents expected that the way the measures were implemented leads to success. While the first component reflected a quite strong identification with the remedial measures, the second component (= way of implementation) seems comparatively minor to parents. It is conceivable that parents were less concerned with whether the remedial measure was actually conducive to learning and compensated for learning losses than with the fact that their children received additional support (during non-school hours, holidays). In other words, parents may primarily seek for additional childcare, “irrespective” of the quality of the implementation, which seems subordinately important to parents.

### Limitations and implications for future research

Firstly, our study represents a cross-sectional study; hence, no causal statements can be made. Nevertheless, cross-sectional studies can provide meaningful insights into the possible longitudinal relations of variables if statistical analyses are rooted in solid theoretical assumptions about predictors and outcomes and if central control variables are modelled. In the present study, we claim both. Even so, future studies should make use of longitudinal data to allow for more causal inferences.

Secondly, from a theoretical point of view, due to the early stage of our study, it was not possible to consider teachers’ and school principals’ recommendations to parents whether their child should attend remedial measures. Additionally, as we opted for a parent survey, we could not assess students’ willingness to attend remedial measures. Future studies should, therefore, incorporate this information into updated predictive models to yield a clearer picture of the underlying mechanisms. Moreover, future studies may not only investigate parents’ intention to use remedial measures, but actual participation.

### Implications for educational policy

Our study revealed a consistent but complex pattern of mechanisms involved in explaining parental intentions to use remedial measures. The pattern is consistent because it is almost identical for all four measures. The pattern is complex because beyond parents’ perception of the quality of distance learning and their child’s engagement, parental values toward remedial interventions emerged as the strongest predictor for the use of these interventions. Moreover, small indirect effects of household income and language spoken at home via parental values on the use of remedial measures were observed. These findings suggest the following measures if participation in interventions is to be increased: (1) It seems to be important to highlight and inform parents about the benefits of remedial measures like summer schools for their children. In particular, it should be emphasised that these measures can be useful even if the school of their children has succeeded in implementing very high-quality distance learning and (2) even if their children belong to the hardworking ones. (3) Educational policy should invest effort in promoting remedial offers among socioeconomically disadvantaged parents and families speaking a non-German language at home. Finally, parents should be informed with respect to expected outcomes of these measures.

## Supplementary Information

The supplementary material contains information with respect to a model adaption due to revision, and with respect to additional interaction analyses due to revision

## Appendix

**Table 4:** Results

	Summer School (REM1)		Tutoring during the Semester Break (REM2)		Tutoring during the Easter Break (REM3)		Additional Tutoring (REM4)	
*Path coefficients*	MLSMV		MLSMV		MLSMV		MLSMV	
	Std. Estimate	*p*-value	Std. Estimate	*p*-value	Std. Estimate	*p*-value	Std. Estimate	*p*-value
*a‑paths (dependent variable: expectancy)*								
Diligence	**−0.095**	0.012	**−0.094**	0.014	**−0.095**	0.013	**−0.095**	0.012
Distance Learning	**0.468**	< 0.001	**0.467**	< 0.001	**0.468**	< 0.001	**0.468**	< 0.001
Stress	**−0.131**	0.002	**−0.134**	0.001	**−0.132**	0.002	**−0.131**	0.002
Qualification	0.066	0.059	0.068	0.052	0.067	0.057	0.066	0.058
Income	0.048	0.157	0.049	0.155	0.048	0.157	0.048	0.157
Language	−0.040	0.297	−0.041	0.290	−0.040	0.294	−0.040	0.295
Partner	−0.029	0.296	−0.029	0.296	−0.029	0.297	−0.029	0.297
*a‑paths (dv: value)*								
Diligence	−0.026	0.327	−0.026	0.323	−0.026	0.326	−0.026	0.327
Distance Learning	0.030	0.367	0.029	0.368	0.029	0.368	0.030	0.368
Stress	0.031	0.238	0.031	0.237	0.031	0.238	0.031	0.239
Qualification	−0.020	0.405	−0.020	0.408	−0.020	0.406	−0.020	0.406
Income	**0.149**	< 0.001	**0.149**	< 0.001	**0.149**	< 0.001	**0.149**	< 0.001
Language	**−0.081**	0.019	**−0.081**	0.019	**−0.081**	0.019	**−0.081**	0.019
Partner	0.015	0.512	0.015	0.510	0.015	0.512	0.015	0.511
*b‑paths (dv: REM)*								
Expectancy	−0.018	0.763	0.055	0.368	−0.001	0.988	−0.035	0.584
Value	**0.234**	< 0.001	**0.256**	< 0.001	**0.141**	< 0.001	**0.143**	< 0.001
*c’-paths (dv: REM)*								
Diligence	**0.110**	< 0.001	**0.145**	< 0.001	**0.078**	0.006	**0.070**	0.016
Distance Learning	**−0.152**	< 0.001	**−0.202**	< 0.001	**−0.151**	0.001	**−0.138**	0.002
Stress	0.039	0.185	**0.063**	0.029	−0.022	0.473	−0.006	0.850
Qualification	−0.016	0.557	**−0.099**	< 0.001	**−0.136**	< 0.001	**−0.119**	< 0.001
Income	0.039	0.202	−0.011	0.707	0.018	0.575	0.010	0.754
Language	0.048	0.096	**0.093**	0.002	**0.095**	0.001	**0.072**	0.016
Partner	0.029	0.214	0.004	0.861	0.035	0.125	0.025	0.275

*REM* remedial measure. *Diligence* Scholastic diligence, *Distance Learning* Quality of distance learning, *Stress* Parental stress, *Qualification* Parents’ highest educational level, *Income* Monthly net household income, *Language* Language spoken at home, *Partner* Single-parent household status

**Table 5:** Full correlation matrix of the study variables

	Monthly net household income	Language spoken at home	Parents’ highest educational level	Single-parent household status	Diligence 1	Diligence 2	Distance Learning 1	Distance Learning 2	Stress 1	Stress 2	Expectancy 1	Expectancy 2	Value 1	Value 2	Value 3	Summer School	Additional Tutoring	Tutoring during the Semester Break	Tutoring during the Easter Break
	1	2	3	4	5	6	7	8	9	10	11	12	13	14	15	16	17	18	19
*Socioeconomic Background*																			
1	–	–	–	–	–	–	–	–	–	–	–	–	–	–	–	–	–	–	–
2	−0.057^**^	–	–	–	–	–	–	–	–	–	–	–	–	–	–	–	–	–	–
3	−0.350^**^	−0.021	–	–	–	–	–	–	–	–	–	–	–	–	–	–	–	–	–
4	−0.333^**^	0.046^**^	0.081^**^	–	–	–	–	–	–	–	–	–	–	–	–	–	–	–	–
*Diligence*																			
5	0.001	−0.010	−0.002	0.041*	–	–	–	–	–	–	–	–	–	–	–	–	–	–	–
6	−0.018	0.002	0.003	0.057**	0.779**	–	–	–	–	–	–	–	–	–	–	–	–	–	–
*Distance Learning*																			
7	0.047*	−0.023	−0.074**	−0.045*	−0.244**	−0.246**	–	–	–	–	–	–	–	–	–	–	–	–	–
8	0.025	−0.011	−0.045**	−0.031	−0.162**	−0.161**	0.570**	–	–	–	–	–	–	–	–	–	–	–	–
*Stress*																			
9	−0.037*	0.019	−0.045**	0.009	0.132**	0.146**	−0.256**	−0.171**	–	–	–	–	–	–	–	–	–	–	–
10	−0.099**	0.017	0.008	0.062**	0.181**	0.204**	−0.312**	−0.211**	0.755**	–	–	–	–	–	–	–	–	–	–
*Expectancy*																			
11	0.007	−0.036	−0.058**	−0.054**	−0.180**	−0.176**	0.259**	0.219**	−0.092**	−0.138**	–	–	–	–	–	–	–	–	–
12	0.089**	−0.039*	−0.119**	−0.041*	−0.066**	−0.069**	0.208**	0.193**	−0.133**	−0.161**	0.276**	–	–	–	–	–	–	–	–
*Value*																			
13	0.109**	−0.074**	−0.026	−0.016	−0.026	−0.038*	0.035*	0.036*	0.044**	0.012	0.034	0.134**	–	–	–	–	–	–	–
14	0.090**	−0.044**	−0.008	−0.009	−0.003	−0.018	0.000	0.025	0.023	0.010	−0.009	0.117**	0.660**	–	–	–	–	–	–
15	0.097**	−0.054**	−0.011	0.003	−0.011	−0.029	−0.011	0.008	0.030	0.030	−0.014	0.121**	0.732**	0.799**	–	–	–	–	–
*Remedial Measures*																			
16	0.000	−0.027	−0.012	−0.043*	−0.134**	−0.146**	0.166**	0.144**	−0.101**	−0.126**	0.196**	−0.056**	−0.141**	−0.167**	−0.187**	–	–	–	–
17	0.052*	−0.052**	−0.118**	−0.036	−0.193**	−0.201**	0.218**	0.152**	−0.139**	−0.164**	0.097**	−0.003	−0.178**	−0.167**	−0.237**	0.542**	–	–	–
18	0.094**	−0.066**	−0.115**	−0.072**	−0.094**	−0.106**	0.156**	0.126**	−0.043*	−0.076**	0.204**	−0.034	−0.072**	−0.097**	−0.123**	0.658**	0.499**	–	–
19	0.074**	−0.046*	−0.097**	−0.067**	−0.107**	−0.110**	0.159**	0.128**	−0.052**	−0.087**	0.213**	−0.030	−0.078**	−0.096**	−0.122**	0.652**	0.499**	0.859**	–

### Differential effects of gender?

We thank Reviewer #1 for redirecting our attention to possible gender effects. Thus, we did multiple group analyses to investigate differences between mothers and fathers with regard to the mechanisms underlying the decision-making investigated in the present study. Findings (Table 6) show that the models with fixed loadings (= metric measurement invariance) and fixed regression paths have significant better model fits. That is, the assumption of different underlying decision-making processes between mothers and fathers is not confirmed (see the better model fit indices for the model with equal/fixed coefficients across the two sex-groups in Table 6). Rather, the data point out that the relations found in the overall model is true for both subgroups.

**Table 6:** Multiple group analyses investigating gender differences

Model		N	#par	χ^2^	df	p	χ^2^/df	CFI	TLI	RMSEA	WRMR	abs (∆χ^2^)	abs (∆df)	p
REM1	Free	3516	177	288.504	127	< 0.001	2.270	0.955	0.915	0.027	0.929	–	–	–
REM1	Fixed	3516	150	224.877	154	< 0.001	1.460	0.980	0.969	0.016	1.008	63.627	27	1.00
REM2	Free	3516	177	236.104	127	0.003	1.860	0.970	0.943	0.022	0.826	–	–	–
REM2	Fixed	3516	150	195.725	154	0.002	1.270	0.989	0.982	0.012	0.910	40.379	27	0.95
REM3	Free	3516	177	287.432	127	0.005	2.260	0.954	0.913	0.027	0.929	–	–	–
REM3	Fixed	3516	150	219.847	154	0.004	1.430	0.981	0.971	0.016	0.996	67.585	27	1.00
REM4	Free	3516	177	277.269	127	0.007	2.180	0.957	0.919	0.026	0.908	–	–	–
REM4	Fixed	3516	150	214.591	154	0.006	1.390	0.983	0.973	0.015	0.977	62.678	27	1.00

*REM* remedial measure, *N* sample size, *Par* Number of parameters estimated, *χ*^*2*^ Chi square value, *df* degrees of freedom, *p* p value, *CFI* Bentler’s comparative fit index, *TLI* Tucker-Lewis index, *RMSEA* root mean square error of approximation, *WRMR* weighted root mean square residual, *R*^*2*^ r square

## Footnotes

1 https://www.bmbwf.gv.at/Ministerium/Presse/20210125.html.

2 For a summary see https://educationendowmentfoundation.org.uk/evidence-summaries/teaching-learning-toolkit/summer-schools/.
