# Supplementary material for: Educational inequality and COVID-19: Who takes advantage of summer schools and other remedial measures? A latent mediation model analysis based on representative data from Austrian parents of school-aged children
Source: Z Bild Forsch. 2022 Sep 16;12(2):407–36. doi: 10.1007/s35834-022-00356-4 (PMC9483411; doi:10.1007/s35834-022-00356-4)
Supplement: Supplementary file 1 — The supplementary material contains information with respect to a model adaption due to revision, and with respect to additional interaction analyses due to revision [file 35834_2022_356_MOESM1_ESM.docx]

**Supplement Material**

*Model adaption due to revision*

To obtain a satisfying model fit, i.e., WRMR values below 1, our original analytical model was re-specified as follows (see also the original and final item list below):

- The indicator “How would you rate your child/youth's learning environment at home during the period of the corona-related school closures in January 2021, e.g., in terms of available computers or space to work?” was removed from the latent factor “quality of distance learning” as this item showed high cross-loadings on the SES factor and the factor “parental stress”.
- The same is true for the item “To meet the obligations, I also have to work at night.” which was used as indicator of the latent factor “parental stress”.
- Regarding the latent factor “scholastic performance”, 3 of the 5 indicators were removed to obtain a narrower construct that assesses students’ consciousness (My youngest school child is busy. My youngest school child is conscientious.). The 3 removed items asked for students’ grades in German, Math, and English and thus showed high error covariation. Hence, we decided to remove them and renamed the latent factor to “scholastic diligence”.

After these modifications of the analytic model the WRMR values improved but were still around 1.2 for the 4 different models of the present paper. Modification indices as provided by Mplus pointed to residual covariances between the indicators of the latent factors “expectancy” and “value”. Although the two latent factors did not significantly correlate with each other, the residuals of the value indicator correlated with each of the residuals of the expectancy indicators by around beta = +/-.200. These error correlations reflect the following: When controlling for parents' general expectation of success of measures and value of measures, parents who are in favor of remedial measures are more likely (1) to think that student teachers' give sufficient consideration to the needs of their child and (2) to think that 2 additional remedial hours are insufficient. Since this seems plausible, we freely estimated these error covariances in the revised model.

**Table 7**. Final Item List

| ***Item*** | ***Description*** |
| --- | --- |
| ***Final item list*** | |
| chil2 | My youngest school child is hardworking. |
| chil3 | My youngest school child is conscientious. |
| parent2 | I hardly have any time for myself anymore. |
| parent3 | I am at my limit. |
| dist2 | In general, how high do you rate the quality of your child's distance education during the corona-related school closings in January 2021? |
| dist3 | Overall, how satisfied are you with the activities your child's school has conducted during the corona-related school closings in January 2021? |
| att1 | Thinking about your child, do you think 2 hours of additional tuition per school week is sufficient? |
| att2 | It is planned by the government that the additional learning support during the vacations will be mainly provided by student teachers. Do you think that they would be able to take sufficient account of your child's needs? |
| val1 | Are you for or against the implementation of additional remedial instruction for lower performing students? |
| val2 | Are you for or against the implementation of German language courses? |
| val3 | Are you for or against the implementation of remedial math courses? |
| ***Items excluded due to revision*** | |
| ger | What are your child's most frequent grades in German? |
| eng | What are your child's most frequent grades in English? |
| math | What are your child's most frequent grades in Math? |
| parent1 | To meet the obligations, I also have to work at night. |
| dist1 | How would you rate your child/youth's learning environment at home during the period of the corona-related school closures in January 2021, e.g., in terms of available computers or space to work? |

*Additional interaction analyses due to revision*

From a theoretical point of view, i.e., according to the expectancy-value framework, an interaction effect between the expectancy and value components is likely. Parents who score high on the expectancy AND value component should be more likely to make use of the remedial measures than parents who score high on only one of the two components. That is, parents who think that the remedial measures will be successful AND who have positive attitudes towards the measures are more likely to also respond affirming to the question on whether they will make use of the remedial measures. Hence, we assume that there is an additional interaction effect beyond the main effects.

To test for the interaction effect, we modelled a latent interaction between the expectancy and value components using the XWITH command in Mplus. However, for three of four models the results indicated non-significant interaction coefficients (REM1: std. beta = -.042, p = .155; REM3: -.023, p = .448; REM4: -.056, p = .067). Thus, the interaction hypothesis is rejected. Only the interaction effect in the “additional tutoring”-model was statistically significant (REM2: -.075, p = .007). However, the effect was negative and thus conflicting with our assumption. That is, parents who think that the remedial measures will be successful AND who have positive attitudes towards the measures are *less* likely to make use of additional tutoring.

From a content-specific point of view, one could explain missing interaction effects as follows. When looking at the item text:

- items of the value component ask whether parents are in favor or against the implementation of remedial measures,
- the items of the expectancy component ask whether parents expect that the way the measures are implemented leads to success.

While the first component reflects a quite strong identification with the remedial measures, the second component (= way of implementation) seems comparatively minor to parents. It is conceivable that parents are less concerned with whether the remedial measure is actually conducive to learning and compensates for learning losses than with the fact that their children receive additional support (during non-school hours, holidays). In other words, parents may primarily seek for additional childcare, “irrespective” of the quality of the implementation, which seems subordinately important to parents.
